# Inhibitory Effects of *Origanum vulgare* Essential Oil on *Mycogone perniciosa* Growth in *Agaricus bisporus* Cultivation

**DOI:** 10.3390/jof11070515

**Published:** 2025-07-09

**Authors:** Jasmina Glamočlija, Marija Ivanov, Marina Soković, Ana Ćirić, Slavica Ninković, Danijela Mišić, Ivanka Milenković, Dejan Stojković

**Affiliations:** 1 Institute for Biological Research “Siniša Stanković”—National Institute of the Republic of Serbia, University of Belgrade, Bulevar Despota Stefana 142, 11108 Belgrade, Serbia; marija.smiljkovic@ibiss.bg.ac.rs (M.I.); mris@ibiss.bg.ac.rs (M.S.); rancic@ibiss.bg.ac.rs (A.Ć.); slavica@ibiss.bg.ac.rs (S.N.); dmisic@ibiss.bg.ac.rs (D.M.); dejanbio@ibiss.bg.ac.rs (D.S.); 2 Ekofungi, Zrenjaninski Put, Padinska Skela, 11213 Belgrade, Serbia; ekofungi@gmail.com

**Keywords:** *Mycogone perniciosa*, RAPD, *Agaricus bisporus*, *Origanum vulgare*, in vitro and in situ fungicidal properties

## Abstract

*Mycogone perniciosa* is the causative agent of wet bubble disease, which induces significant losses in the production of *Agaricus bisporus*, indicating the high importance of the development of novel inhibitory agents. The isolation, identification, and molecular characterization of five isolates of *M. perniciosa* from diseased fruit bodies of *A. bisporus* was done. Moreover, the study evaluated the in vitro and in situ potential of *Origanum vulgare* essential oil (EO) to limit *M. perniciosa* growth and provided chemical characterization of its volatile components. The obtained strains differed phenotypically and according to their molecular characteristics. *O. vulgare* EO has shown more promising antifungal activity than the commercial fungicide Prochloraz-Mn in the microatmospheric method. In the treatment of experimentally induced wet bubble disease on *A. bisporus* in the growing chambers with 2% of *O. vulgare* EO and simultaneous application of spore suspension of mycopathogen, *O. vulgare* EO totally inhibited the growth of *M. perniciosa*. Carvacrol, p-cymene, γ-terpinene, and thymol were dominant constituents of *O. vulgare* EO examined in this study. *O. vulgare* EO has shown promising potential to limit growth of *M. perniciosa* and should be further explored as a novel biofungicide.

## 1. Introduction

Mushrooms are popular as food worldwide due to their pleasant taste and health-beneficial properties associated with their intake [1]. *Agaricus bisporus* is among the most sought-after cultivated mushrooms. It is rich in polysaccharides, like bioactive β-glucans, and associated with numerous beneficial properties [2]. Moreover, this species is abundant in other bioactive mycoconstituents such as phenolic acids, saturated fatty acids, and β-tocopherol [3]. These bioactive molecules, along with the appealing flavor which makes them popular in culinary uses, contribute to the mushroom’s widespread popularity and growing demand for its production. Some studies provide supportive evidence for the cultivation of this species as part of urban agriculture that might provide sustainable solutions for some of the global challenges [4].

Numerous infections pose serious risks to production and quality in the regulated conditions used to cultivate *A. bisporus*. These consist of bacterial, fungal, and, less frequently, viral agents. The most common and harmful of these are fungal pathogens, particularly in the warm, humid environments found in mushroom-growing units. The most prevalent and economically significant pathogens are *Cladobotryum* species (cobweb disease), *Trichoderma aggressivum* (green mold), *Verticillium fungicola* (dry bubble), *Mycogone perniciosa* (causative agent of wet bubble disease), and bacterial pathogens like *Pseudomonas tolaasii* (bacterial blotch) [5].

One of the most common diseases of *A. bisporus* in mushroom farms is wet bubble disease (WBD), caused by the fungus *Hypomyces perniciosus* (formerly *Mycogone perniciosa*) [5]. *M. perniciosa* can easily spread from the initial source of infection by using air currents, water splashes, and phorid and sciarid flies, with its growth being stimulated by metabolites of the growing *A. bisporus* mushroom mycelium. It is noted that the duration from infestation to symptom expression varies from 2–3 to 12 days in most of the cases [6]. Wet bubble disease is the reason for drastic economic losses in button mushroom production, with China, for example, losing about 15–30% of mushroom yields due to this disease [7]. *M. perniciosa* infections are not limited to *A. bisporus* but can affect other species such as *Pleurotus ostreatus*, *P. citrinopileatus*, and *Volvariella volvacea* [8], but also found in the wild *Agaricus arvensis* [9].

There are different fungicides for the control of this disease. However, more recently, some studies report on the problem of resistance being developed to the available protective chemicals [7]. On the contrary, another study [8] found prochloraz-Mn and carbendazim to be effective in inhibiting the growth of *M. perniciosa* and highlighted the low risk for the development of resistance. However, even if the resistance is not in question, some concerns are raised in regard to the application of fungicides. For example, carbendazim interferes with the antioxidant defense system and is teratogenic, mutagenic, and aneugenic. The range of abnormalities induced by this fungicide includes hematological abnormalities, mitotic spindle deformity, endocrine disruption, embryo toxicity, infertility, and others [10].

Fungicides based on plant products have diverse beneficial properties, such as having lower resistance and being environmentally friendly [11]. EOs are especially known for their antifungal activity, with their mechanisms often being related to the interference with cell membrane or cell morphology by EO and its constituents [12]. Among the most studied antifungal EOs is *Origanum vulgare*. *O. vulgare*, oregano, is one of the most used aromatic species, both as a spice and medicinal plant, mainly due to the high content of its EO, with commercial *O. vulgare* being both wild collected and cultivated [13]. This oil can inhibit the growth of different fungal pathogens such as *Botrytis cinerea* [14] and *Candida* spp. [15]. The antifungal activity of *O. vulgare* EO could be attributed to diverse phytochemicals with strong antifungal potential that are present in the oil, such as thymol and carvacrol [16].

Despite the abundant potential of *O. vulgare* EO to reduce growth rates of different fungal pathogens, not much is known regarding its effect on the most common pathogen of button mushrooms, *M. perniciosa*. Furthermore, research on the bioactive properties of *O. vulgare* EO with in situ studies is at the moment scarce, highlighting a significant gap in the current knowledge. Our study aimed to determine the antifungal activity of *O. vulgare* EO towards different strains of *M. perniciosa* isolated from button mushrooms. Furthermore, we have evaluated the efficacy of the oil in the in vitro and in situ infection models. Additionally, chemical characterization of the EO was conducted.

## 2. Materials and Methods

### 2.1. Fungal Strains and Growth Media

Samples of diseased *A. bisporus* fruiting bodies were collected in mushroom farms from Serbia (isolates MPPS (Padinska Skela) and MPR (Ripanj)) and Bosnia and Herzegovina isolate MPS (Sarajevo). On the surface of the fruiting bodies, after 3 days of incubation in a humid chamber, a white mycelium developed. The mycelia were isolated on a medium MA (Torlak, Belgrade, Serbia) with antibiotics streptomycin (Sigma Aldrich, Saint Louis, MO, USA) in order to eliminate bacteria, and then the pure culture was isolated on PDA (Neogen, Lansig, MI, USA) [17]. The isolates were identified according to the taxonomic criteria described by Brady and Gibson [18]. The two strains that were obtained from Mushroom Experimental Station (Horst, The Netherlands) (MPH1—CBS 815,73; MPH2—CBS 322,52), are from international culture collections Centraalbureau voor Schimmelcultures (CBS, Utrecht, The Netherlands). Pure cultures of the *M. perniciosa* were deposited at the Fungal Collection Unit of the Institute for Biological Research “Siniša Stanković”—National Institute of the Republic of Serbia; University of Belgrade. The mycopathogens were maintained on potato dextrose agar (PDA). The cultures were stored at 4 °C and subcultured once a month [17].

### 2.2. DNA Extraction and RAPD-PCR Analysis

#### 2.2.1. DNA Extraction

To produce fungal biomass for DNA extraction, the *Mycogone* isolates were grown for 7 days at 25 °C in stationary conditions in Erlenmeyer flasks (500 mL) containing 200 mL liquid potato dextrose yeast medium (PDY) (Torlak, Belgrade, Serbia), harvested by filtration, then washed twice with sterile water and dried at room temperature [19].

DNA extraction was carried out using the methods of Mello et al. [20] with slight modifications. Approximately 250–300 mg of tissue was crushed in the presence of liquid nitrogen and homogenized with 700 µL extraction buffer (100 mM Tris–HCl pH 8.0, 1.4 M NaCl, 20 mM EDTA, 2% CTAB, 0.2% β-mercaptoethanol, Thermo Scientific, Waltham, MA, USA). All samples were incubated at 65 °C for 1 h, centrifuged for 5 min at 13,000 rpm, and extracted with 1 volume of chloroform/isoamyl alcohol (24:1) (Acros Organics, Geel, Belgium). Nucleic acid in the supernatant was precipitated with 1.5 volumes of isopropanol (Fisher Scientific, Waltham, MA, USA) and centrifuged for 15 min at 13,000 rpm for each extraction. Samples were incubated at −20 °C for 30 min, centrifuged at 13,000 rpm for 30 min, and the pellet washed with 1 mL of 70% ethanol (Zorka, Šabac, Serbia), well dried, and resuspended in 200 µL of 1× TE buffer (10 mM Tris–HCl pH 8.0, 1 mM EDTA) at 4 °C during the night. DNA is treated with RNase (1 mg/mL) (Acros Organics, Geel, Belgium). To 100 µL of DNA solution, 5 µL of RNase was added and incubated at 37 °C for 30 min. The final DNA concentration was estimated by agarose gel electrophoresis at 1.5% agarose gel in 1× TBE buffer (45 mM Tris-borate, 1 mM EDTA, pH 8.0).

#### 2.2.2. RAPD-PCR Analyses

For RAPD analysis [19], primers 268 (AGGCCGCTTA), 266 (CCACTCACCG), and 280 (CTGGGAGTGG) supplied by Applied Biosystems UK were used. In a total volume of 25 µL (10 ng DNA, reaction buffer 10× (Applied Biosystems UK, Warrington, UK), 2.5 mM dNTP MIX (Applied Biosystems UK), 5 μM decamer primers, 5 U/μL Ampli Taq Gold (Applied Biosystems, Warrington, UK), and 2 mM MgCl_2_. Amplification was performed in a Genius-Techne thermal cycler (Cambridge, UK) as follows: initial denaturation for 2 min at 94 °C, then 35 cycles of 1 min at 94 °C, 2 min at 37 °C to 20 °C (35 cycles—in each cycle temperature is lower for 0.5 °C), and 2 min at 72 °C, followed by a final extension for 5 min at 72 °C. Amplification products were separated by electrophoresis on 1.5% agarose gel in 1× TBE buffer, stained with ethidium bromide, and visualized under UV light.

### 2.3. GC-MS Analysis of O. vulgare Volatile Compounds

*O. vulgare* EO was produced by the distillation company Sanicula Co., Ltd., Paraćin, Serbia. *O. vulgare* EO was dissolved in absolute ethanol (1:99), and the sample was transferred to a 2 mL vial for GC-MS analysis. Analysis of volatile compounds was performed on an Agilent 8890/5977BMSD/FID GC-MS system using an Agilent (Santa Clara, CA, USA) HP-INNOWax (PEG stationary phase) column with a length of 30 cm, an internal diameter of 250 μm, and a coating thickness of 0.25 μm. From each sample, 1 μL was injected in a splitless flow mode at the injector temperature of 250 °C. Helium 5.0 was used as carrier gas at a flow rate of 0.8 mL/min. The oven temperature program included an initial temperature of 65 °C, at a rate of 3 °C/min, ramping up to 220 °C and holding for 15 min. The mass detector temperature was set at 150 °C, and the transfer line temperature was 250 °C. Ionization was achieved through electron impact (EI) at 70 eV. To establish the retention index, a mixture of C10-C40 normal alkanes (Sigma, St. Louis, MO, USA) was used in conjunction with AMDIS ver. 2.73 (National Institute of Standards and Technology (NIST), Gaithersburg, MD, USA). The FID detector operated at a temperature of 300 °C. Compounds were identified by comparing the Kovach retention index and mass spectrum with those of standard substances and mass spectral data from the National Institute of Standards and Technology (NIST MS Search, Version 2.4) libraries, the Robert Adams library, and pertinent literature data. MSD ChemStation software (F.01.03.2357, Agilent Technologies, Inc., Santa Clara, CA, USA) was used to integrate and determine the peak areas of individual volatile compounds. The amounts of individual components were expressed as a percentage of the total chromatogram area based on data from the FID detector [21].

### 2.4. In Vitro Antifungal Activity

The modified microatmosphere method [22] was used for the investigation of the antifungal activity of *O. vulgare* EO. Petri plates measuring 50 mm were filled with 10 mL of PDA medium and then were seeded with a small amount of 7-day-old mycelium culture of the *M. perniciosa* strains. The Petri dishes were then inverted, and the determined amount (0.001–20 μL/mL) of pure oil impregnated on sterile filter paper discs (6 mm in diameter) was deposited on the inverted lid. Prochloraz-Mn, (((1-N-propyl-N-2(2,4,6-trichlorophenoxy) ethyl) carbomoyl imidazole + Manganese)) (“Mirage45-EC”, Makhteshim Chemical Works, Beer-Sheva, Israel) was used as a positive control (1 mg/mL; 5–>50 μL/disc). Minimal inhibitory quantities (MIQ) and minimal fungicidal quantities (MFQ) of EOs were noted upon 7 days of incubation at 25 °C.

### 2.5. In Situ Antifungal Activity

The experiments were carried out in the cultivation chamber in bags, 40 × 60 × 25 cm, filled with compost phase III spawned with A15 mushroom strain from a commercial mushroom compost yard (Company EKOFUNGI, Padinska skela, Serbia). The surface of the compost was covered with an 8 cm layer of black peat. The conditions in the mushroom house chamber were fully controlled for routine production; that is, the temperature was 24 °C, the carbon dioxide concentration was 3000 mg/L, and the relative humidity was 95%. After 8 days, when the mycelium reached the surface of the casing, the temperature and carbon dioxide concentrations were lowered to 18 °C and to 800 mg/L, respectively.

To treat experimentally induced WBD in situ, we tested the antifungal activity of *O. vulgare* EO when applied in casing soil in different ways. Yield performance of *A. bisporus* fruiting bodies was measured under different treatment conditions: 1—Untreated control; 2—Treated with prochloraz-Mn; 3—Treated with 2% *O. vulgare* EO; 4—Inoculated with *M. perniciosa*; 5—2.0% oil added to casing soil, and 4 h later the spore suspension of 1.2 × 10^6^ spors/mL was applied and put on the compost; 6—2% *O. vulgare* EO added simultaneously with a suspension of 1.2 × 10^6^ spors/mL in casing soil and put on the compost layer immediately. The suspensions of the *M. perniciosa* spores were prepared on the day of *A. bisporus* inoculation. Conidia were prepared by washing the surface of agar slants (PDA) 2-week-old pure cultures of *M. perniciosa* with nonpyrogenic sterile physiological saline with Tween 40 (Fisher Bioreagents, Pittsburgh, PA, USA). Obtained conidia were counted using an improved Neubauer hemocytometer (Neubauer chamber, Paul Marienfeld, England) and adjusted to the desired concentration. The occurrence of fruiting of *A. bisporus* was monitored for 25 days, the appearance and number of fruiting bodies, and the appearance of disease symptoms.

### 2.6. Statistical Analysis

Experiments were performed in three replicates, and statistical analysis was calculated by two-way ANOVA (GraphPad Prism 9.0.0).

## 3. Results and Discussion

### 3.1. The Isolation of M. perniciosa Isolates

**Figure 1:**
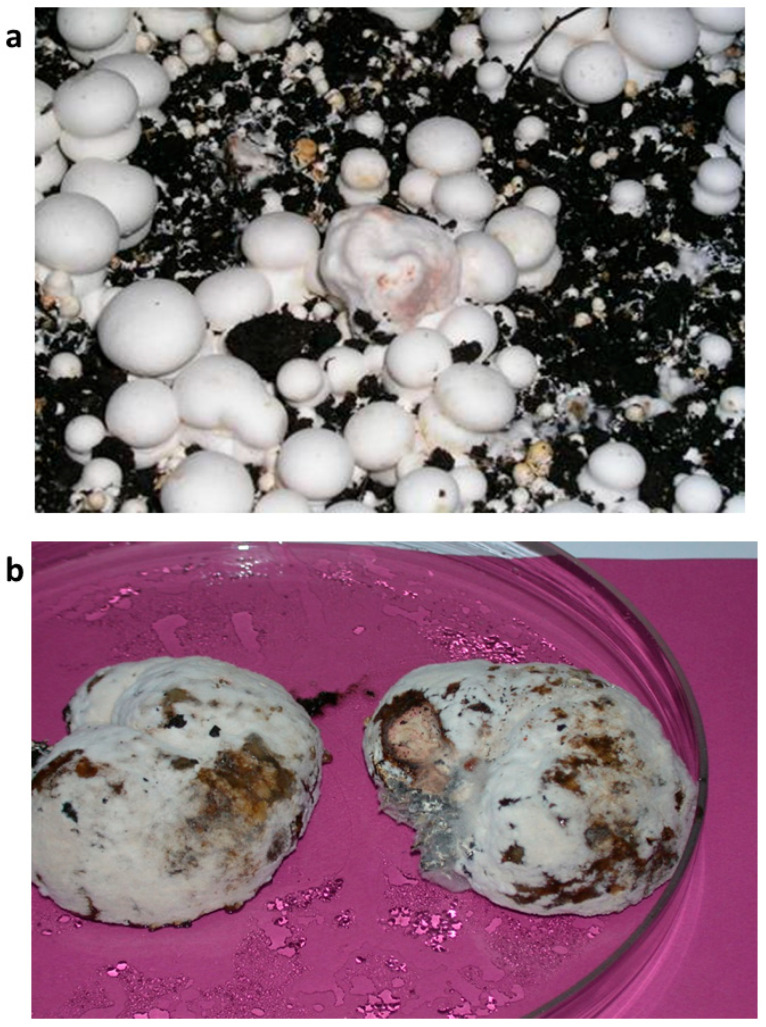
Fruiting bodies of *A. bisporus* with symptoms of WBD, fresh (**a**) and on the third day in a humid chamber (**b**).

The isolation and identification of isolates of *M. perniciosa* from diseased fruiting bodies of white button mushrooms from mushroom units in Serbia and Bosnia and Herzegovina were made (Figure 1).

**Figure 2:**
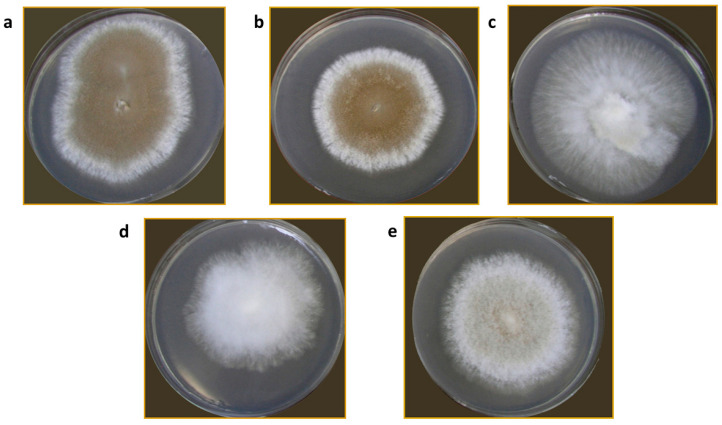
*M. perniciosa* isolates on PDA plates: MPPS (**a**); MPR (**b**); MPS (**c**); MPH1 (**d**); MPH2 (**e**).

Colony characteristics of all isolates varied. The colonies were very regular in growth with either dense aerial mycelium MPH2, MPPS, and MPR, or sparse MPH1. Only the MPS isolate had a colony, which grew in compact and aerial sectors, irregular in growth. Colony color, which to some extent indicates the production of aleuriospores, varied from white for the MPS isolate to dark brown for the MPPS isolate (Figure 2).

Mycopathogenic and different competing types of microorganisms inhabit the cover of *A. bisporus* mycelium, including species that cause WBD and *M. perniciosa* [23]. The presented isolation of different strains of *M. perniciosa* from *A. bisporus* collected from mushroom farms in different European countries confirms the widespread presence of the causal agent of WBD across Europe [11,24]. The variability in colony morphology among different isolates indicates potential diversity within *M. perniciosa* strains from different origins. This phenotypic diversity observed on PDA plates might be the consequence of genetic variability that is further explored in this manuscript. Additionally, two isolates from Serbia, MPPS and MPR, have shown similar growth patterns. Moreover, in the previous study, authors have implied different growth rates and characteristics on PDA of strains from different origins [25]. The study by Li et al. [8] pointed out the possible connection between the color of the colonies and the strains pathogenicity, which is another feature that should be explored in the future.

### 3.2. RAPD-PCR Analyses of M. perniciosa Isolates

**Figure 3:**
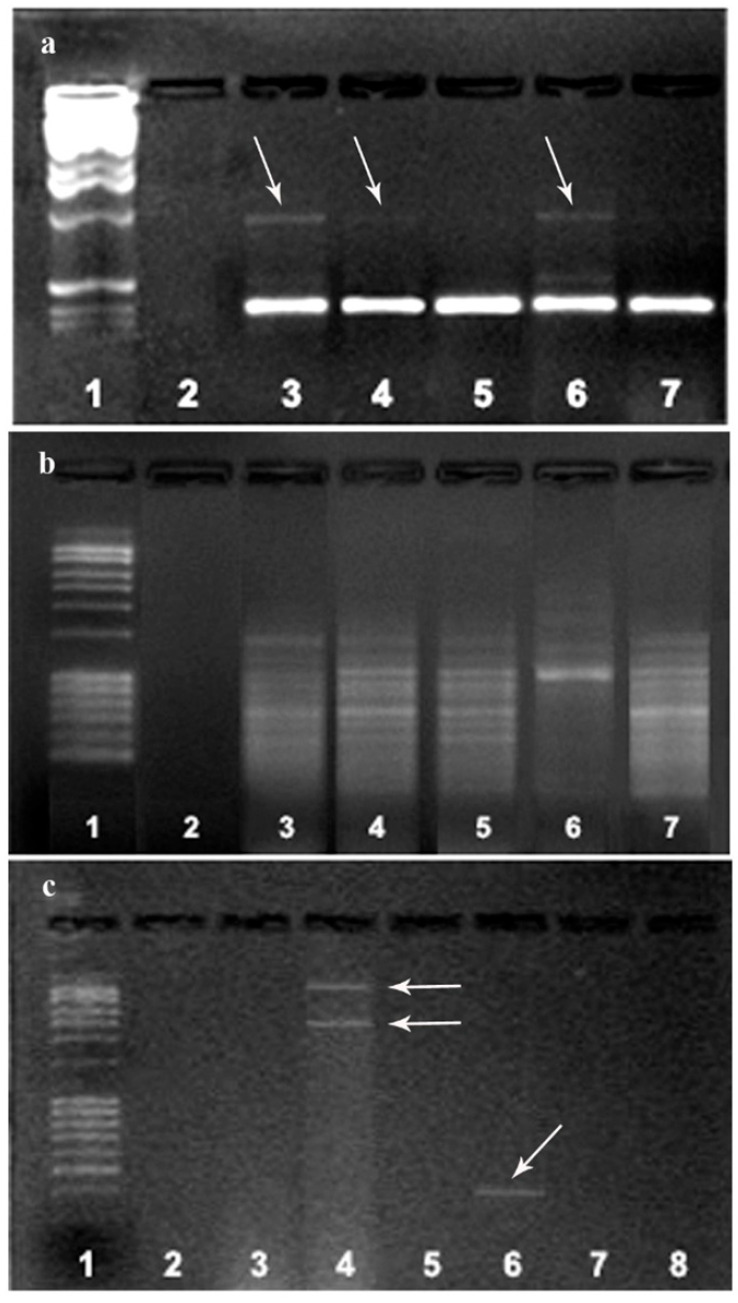
Comparison of the RAPD patterns of *M. perniciosa* isolates: RAPD fragments obtained with primer 268 (**a**) and 266 (**b**): marker (line 1); negative control (line 2); MPH1 (line 3); MPH2 (line 4); MPS (line 5); MPPS (line 6); MPR (line 7); (**c**) RAPD fragments obtained with primer 280: marker (line 1); MPH1 (line 2); MPH2 (line 3); MPPS (line 4); MPS (line 5); MPR (line 6); negative controls (lines 7, 8). The arrows represent the detected polymorphism between the tested isolates MPH1, MPH2 and MPPS in relation to the other isolates with primer 268 (**a**) and two fragments obtained in the MPPS isolate that did not occur in of another isolate. Fragments are different sizes are also present in MPR isolates with primer 266 (**c**).

The obtained *M. perniciosa* isolates were molecularly characterized by using RAPD markers. RAPD profiles of MPH1, MPH2, and MPPS showed relative heterogeneity (Figure 3a). Primer 266 did not reveal any intra-isolate variability among major bands (Figure 3b), whereas primer 280 showed a high degree of polymorphism between isolates from Serbia (MPPS and MPR) and the other two groups (Figure 3c).

According to the morpho-physiological characteristics and RAPD analyses of five *M. perniciosa* isolates, isolates from Serbia were similar; the isolates from the Netherlands showed mutually similar characteristics, but they were different from isolates from Serbia. The isolate from Bosnia and Herzegovina is different from these two groups. For further tests, the MPS isolate was used.

It seems that the combination of morpho-physiological traits previously observed and RAPD patterns supports the classification of isolates according to their geographical origin. This might have potential implications for disease management strategies in different regions. Previously, the study by Li et al. [8] found no evidence of an association between the genetic diversity and the geographical origin of the isolates from different regions of China, suggesting that further research on more strains and additional locations is needed to provide strong evidence. On the other hand, genetic diversity of 38 *M. perniciosa* isolates from different areas in China was analyzed by sequence-related amplification polymorphism (SRAP), with the strains from the same provinces being clustered in the same clade [25]. The limits of this evaluation of diversity are limited sample size and geographical coverage, future studies should include additional regions and additional samples from the same region in order to provide further evidence for the link between the origin of the strains and their characteristics. Additionally, whole-genome sequencing of the strains could reveal other differences among them and could provide a more detailed understanding of their evolutionary relationships, virulence, and genetic adaptations to specific environmental conditions.

### 3.3. EO Composition

The volatile profile of *O. vulgare* EO is characterized by a complex mixture of monoterpene and sesquiterpene molecules (Table 1). Carvacrol (50.44%) and thymol (7.55%), both phenolic monoterpenes, are the main representatives of the oxygenated monoterpenes (62.04%) chemical class. These substances have a hydroxylated aromatic ring in common, which gives them a higher polarity than other monoterpenes. *p*-Cymene (17.54%) and γ-terpinene (8.52%) are the most prevalent monoterpene hydrocarbons that constitute 33.43% of the *O. vulgare* EO volatile content. Other detected monoterpenes were (+)-4-carene (2.52%), a bicyclic monoterpene with a fused cyclopropane-cyclohexane structure, and β-myrcene (2.16%), a linear acyclic monoterpene with a conjugated diene system. Caryophyllene (0.88%) is the most prevalent of the sesquiterpene hydrocarbons, which make up 1.69% of the overall composition. Our previous study [26] on *O. vulgare* EO has also demonstrated the prevalence of carvacrol as the main constituent of EO. In comparison to our previous investigation, the current study brings more identified compounds within the volatile profile of *O. vulgare*. Comparative study of numerous EOs from Europe indicated that they are rich in monoterpenes such as sabinene, myrcene, *p*-cymene, 1,8-cineole, β-ocimene, γ-terpinene, sabinene hydrate, linalool, α-terpineol, carvacrol methyl ether, linalyl acetate, thymol, and carvacrol. Sesquiterpenes such as β-caryophyllene, germacrene D, germacrene D-4-ol, spathulenol, caryophyllene oxide, and oplopanone were also notable constituents [27]. Subsequent studies are needed to reveal the possible impact of storage duration and different conditions on the chemical composition of EO, since it may affect its constituents and subsequent bioactive properties.

### 3.4. In Vitro Antifungal Activity of O. vulgare EO

Antifungal activity of *O. vulgare* EO towards different *M. perniciosa* strains was strong and highlighted with MIQ in the range of 0.1–2.0 µL/disc and MFQ in the range of 0.5–5.0 µL/disc (Table 2). The oil of *O. vulgare* possessed the strongest antifungal activity against *M. perniciosa* MPS with a minimal inhibitory quantity of 0.1 μL/disc and a minimal fungicidal quantity of 0.5 μL/disc (Table 2). The fungicide prochloraz-Mn, which we used as a control, showed much lower antifungal activity than *O. vulgare* EO towards all strains tested, with MIQ 5.0 μL/disc and MFQ 50.0 μL/disc or ≥50.0 μL/disc. It was thought that vapor treatment would be very effective against *M. perniciosa,* allowing the use of only a limited amount of EO. Despite similar sensitivity of the examined fungal strains to prochloraz-Mn treatment, their susceptibility to *O. vulgare* EO varied greatly. We can observe a similar pattern of EO sensitivity based on the strain’s geographical origin, with the highest sensitivity recorded for the strain from Bosnia and Herzegovina (MPS, MIQ 0.1 µL/disc), followed by two strains from the Netherlands (MPH1 and MPH2 with MIQ 0.3 and 0.2 µL/disc, respectively), while the highest resistance can be attributed to the two strains from Serbia (MPPS and MPR with MIQ 1.0 and 2.0 µL/disc, respectively).

*O. vulgare* EO is a well-known antifungal agent. Its ability to limit the growth of microbial pathogens has been previously explored towards *Botrytis cinerea*, where the in vivo vapor contact assay suggested that oil had antifungal activity at 250 mg/L and it reduced the decay of cherry tomatoes by 96.39% [14]. Its antibacterial potential is also well-known [28], implying the wide antimicrobial application of this natural product. Carvacrol and thymol were found as the dominant constituents of *O. vulgare* EO examined in this study along with the *p*-cymene and γ-terpinene and might be the underlying reason for the observed antifungal activity. The carvacrol antifungal mechanism was investigated previously towards *Candida albicans*, and it involved binding to sterols in the fungal membrane [29]. Furthermore, both thymol and carvacrol inhibit the growth of *B. cinerea* by interfering with membrane permeability and destroying the cell membrane structure [30]. Thymol induced a similar protective effect as the commercial fungicide (Chronos 450 SC) on *A. bisporus* by inhibiting the growth of *M. perniciosa* while at the same time having minimal effect on *A. bisporus* [31]. Antimicrobial properties of *p*-cymene are being linked with its ability to impact the stability of microbial cell membranes [32], while the study of *Bursera morelensis* Ramirez EO found its promising anti-*Candida* potential, possibly due to interaction among the dominant molecules α-pinene and γ-terpinene [33]. Even the compounds found in lower abundances in *O. vulgare* EO (Table 1) have been known as antifungal agents. This is the case with linalool, a compound that inhibits the growth of the pathogenic fungus *Fusarium oxysporum* f. sp. *radicis-lycopersici* by interfering with cell membrane integrity and enhancing levels of reactive oxygen species [34].

The goal of out future research is to link the mechanisms of antifungal activity for the *O. vulgare* EO dominant molecules with the antifungal mode of action for *O. vulgare* towards *M. perniciosa*. Likewise, including additional *M. perniciosa* strains when assessing *O. vulgare* EO antifungal activity would provide compeling evidence for the potential wide application of this natural product and additional connection of the susceptibility to EO with the geographical origin of the strains. Future studies might be focused on elucidating the nature of interaction between *O. vulgare* EO and Prochloraz Mn with the goal of revealing potential synergistic effects that might improve antifungal efficacy while at the same time reducing the dosages of synthetic fungicides.

### 3.5. Investigation of O. vulgare EO In Situ

**Figure 4:**
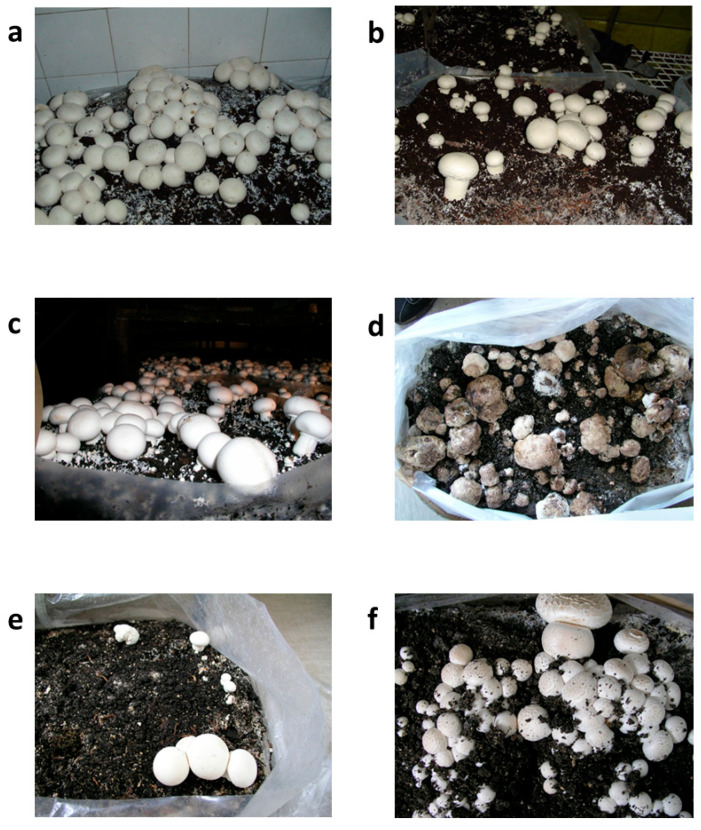
Appearance of *Agaricus bisporus* fruiting bodies after 20 days: (**a**) untreated control; (**b**) treated with prochloraz-Mn; (**c**) treated with 2% *O. vulgare* EO; (**d**) inoculated with the pathogen *Mycogone perniciosa*; (**e**) inoculated with *M. perniciosa* followed by *O. vulgare* EO application 4 h before inoculation; and (**f**) simultaneously inoculated with *M. perniciosa* and treated with *O. vulgare* EO.

Strain *M. perniciosa* MPS was selected for the assessment of in situ sensitivity to *O. vulgare* EO due to its higher sensitivity to the in vitro treatment in comparison with other tested *M. perniciosa* strains. The morphological traits of *A. bisporus* fruiting bodies under different treatment settings after 20 days are depicted in Figure 4. This information offers insight into the course of the disease and the effectiveness of the treatment, with consequences for yield and economic viability. Both the prochloraz-Mn-treated group (Figure 4b) and the untreated control group (Figure 4a) showed completely grown, morphologically normal fruiting bodies, which can be considered as a sign of ideal yield performance conditions. The size, color, and integrity of the fruiting bodies treated with 2% *O. vulgare* EO (Figure 4c) were similar to those in the control groups, indicating that *O. vulgare* EO had no detrimental effects on biomass accumulation or growth. At this point, it is worth noting that in this sample, establishment of primordia was detected at higher levels when compared to the control groups 1 and 2. According to this observation, *O. vulgare* EO maintained yield at levels similar to those of conventional fungicidal treatments when applied as a protective treatment (Table 3).

On the other hand, the *Mycogone perniciosa*-inoculated group (Figure 4d) that received no treatment showed obvious symptoms of infection, such as structural deterioration and tissue softening, which are traits of WBD. Given this morphology, a significant yield loss is anticipated in terms of both marketable quality—a crucial component of commercial mushroom production—and overall biomass.

Fruiting body integrity appeared to be partially intact in the group that received *O. vulgare* EO four hours before pathogen inoculation (Figure 4e), which indicated a less severe case of the disease. Nonetheless, there has still been some deformation or delayed growth, which has led to a slight yield drop (Table 3). However, when early indications of infection are found, this condition may serve as a salvage tactic, possibly averting complete crop loss. Fruiting bodies that received *O. vulgare* EO and *M. perniciosa* inoculation at the same time (Figure 4f) were completely healthy and comparable to the untreated group. The structure’s preservation implies that early intervention or *O. vulgare* EO co-treatment may prevent early pathogen establishment, preserving yields that are more in line with those of untreated or fungicide-treated groups. This preventative measure may provide a natural and efficient way to manage disease, especially in low-chemical or organic production systems.

The data in Table 3 demonstrate how the yield and quality of *Agaricus bisporus* fruiting bodies are affected by *M. perniciosa* infection and subsequent *O. vulgare* EO treatment. All fruiting bodies in the untreated control group were healthy and obtained the highest visual grading score (5), resulting in a total yield of 1.19 kg per bag. Prochloraz’s effectiveness in preserving crop health and appearance was further demonstrated by the slightly greater yield (1.21 kg/bag) and 100% healthy fruiting bodies in the group treated with the fungicide. The fruiting bodies treated with 2% *O. vulgare* EO alone produced the maximum output of any sample (1.24 kg/bag), indicated by a intact and healthy fruiting body and supperior visual appeal. This implies that under typical, uninfected conditions, *O. vulgare* EO may have a minor yield-promoting effect in addition to having no detrimental effects on mushroom development.

The group that was injected with *M. perniciosa* alone (Sample 4) had a significant decrease in yield (0.75 kg/bag), with just 8% of fruiting bodies staying healthy and the lowest visual grading (1), indicating the fungus’s extreme pathogenicity. Partial protection was seen when *O. vulgare* EO was applied four hours before inoculation (Sample 5). The visual grade was lower (3); suggesting that some degree of damage or deformation occurred after treatment, even though the overall yield increased to 0.97 kg/bag and 87% of the fruiting bodies remained healthy. Sample 6 showed the greatest results among the infected groups, with a total output of 1.04 kg/bag, 100% viable fruiting bodies, and the highest visual grade (5) due to simultaneous *M. perniciosa* inoculation and *O. vulgare* EO treatment. These results imply that *O. vulgare* EO might successfully prevent infection and preserve yield and quality when used in conjunction with pathogen exposure, possibly outperforming synthetic fungicides. The information suggests that *O. vulgare* EO is a viable natural remedy for controlling *M. perniciosa* infections in *A. bisporus* production, particularly when used early or in conjunction with pathogen exposure. We have observed a higher percentage of healthy fruiting bodies when *O. vulgare* EO was applied simultaneously with the pathogen, rather than when it was applied prior to infection. On the other hand, the total yield remained similar in both cases. This might be due to the rapid dissipation of active volatile components before pathogen contact when applied too early, with further research needed to confirm this. Moreover, further research might explore the antifungal activity of encapsulated oil, since encapsulation might slow down the release of active volatile components and provide a prolonged bioactive effect [35], which should be additionally studied.

The in situ experiment was based on the effects of one selected *M. perniciosa* strain. Future evaluation should reveal protective effects of *O. vulgare* EO when *A. bisporus* is exposed to additional *M. perniciosa* strains. Also, only two *O. vulgare* EO application timings were evaluated, limiting the understanding of how treatment timing affects infection and its inhibition. Additionally, a larger-scale field trial could provide more firm evidence for the observed protective effect of *O. vulgare* EO on spread of the disease.

A representative and high-stakes model for researching pathogen resistance, sanitation practices, and the effectiveness of biocontrol agents in *A. bisporus* farming is offered by concentrating on *M. perniciosa*. When this pathogen is successfully controlled, it frequently leads to more extensive advancements in disease prevention and cleanliness practices, which can lower the risk of other infections.

Previous investigations of different chemical fungicides found their promising antifungal activity, especially in the case of prochloraz-Mn. On the other hand, the same study indicated the low effect of bio-fungicides based on *Bacillus* strains [36]. It seems that the antifungal activity of EOs is not limited to certain types of pathogens since their inhibiting efficacy is noted towards human pathogens [37], animal pathogens [38], and causes of plant diseases [39]. These natural products should be further explored by including additional EOs in the analysis of their protective effects on *A. bisporus* and by optimizing EO delivery methods.

A number of studies have examined the effect of essential oils on the pathogens of button mushrooms [40]. It has been proven that essential oils not only have a contact effect, but also, by vaporizing, the active agents can block the vegetative growth of pathogens [41].

The in vivo trial using *M. perniciosa*-inoculated casings revealed that the preventative use of lemon, verbena, or thyme oils was able to control the development of the WBD. The two of their main components (nerol and thymol) revealed that none of these treatments were detrimental to the growth of the *A. bisporus* [31].

Our experiment has not included button mushroom mycelia in the tested main components (Carvacrol, *Thymol, p*-Cymene, and γ-Terpinene). The World Health Organization (WHO, 2014) confirmed that thymol and carvacrol residues in food are harmless to consumers as long as they do not exceed 50 mg kg^−1^ [42]. Additionally, *O. vulgare* EO has been regarded as safe in different studies. The study by Llana-Ruiz-Cabello et al. evaluated the effect of *Origanum vulgare* L. virens EO on the rodents during the 90 days application and found no mortality and no treatment-related adverse effects at 200 mg/kg b.w. in Wistar rats [43]. Another study found the IC_50_ value of *O. vulgare* EO towards HEK293 cells, a human embryonic kidney cell line, to be at 310 µg/µL [44]. On the other hand, research on *Zea Mays* seeds found that *O. vulgare* EO has genotoxic activity by affecting both DNA and proteins [45]. The previous findings highlight the need for additional toxicological evaluations of *O. vulgare* EO to fully elucidate its safety profile across different biological systems.

## 4. Conclusions

In this study we have observed both morphological and genetic diversity among *M. perniciosa* strains from different origins, indicating that more detailed studies are needed to explore additional links between colony morphology and genetic characteristics with virulence and susceptibility to fungicides. Taking into account the toxicity of synthetic fungicides, which reach humans through the food chain, there is an increasing need to find alternative fungicidal agents. Current knowledge indicates that plant metabolites show good antifungal activity and can be considered less harmful in comparison with synthetic fungicides. Additionally, we have provided strong evidence for *O. vulgare*’s potential to limit growth of *M. perniciosa* both in vitro and in situ. This natural product, along with its dominant constituent, carvacrol, should be further explored in order to reveal its antifungal mechanism and potential to be applied in basement cultivation of *A. bisporus*, especially in organically certified mushroom production farms.

## Figures and Tables

**Table 1:** Chemical composition (% *v*/*v*) of *O. vulgare* EO.

Compound	Molecular Formula	Retention Time (RT)	Base Peak Area	Composition (%)
*α*-Thujene	C_10_H_16_	6.355799737	301,135.2099	1.48
(+)-Camphene	C_10_H_16_	6.530872701	54,440.09729	0.27
β-Myrcene	C_10_H_16_	6.945883317	438,630.2623	2.16
*δ*^3^-Carene	C_10_H_16_	7.165514349	29,277.81884	0.14
(+)-4-Carene	C_10_H_16_	7.224131263	511,904.0637	2.52
*p*-Cymene	C_10_H_14_	7.301926701	3,565,433.367	**17.54**
(+)-Sylvestrene	C_10_H_16_	7.343570592	113,330.95	0.56
Eucalyptol	C_10_H_18_O	7.384335853	7,675.914505	0.04
β-Ocimene	C_10_H_16_	7.494239672	14,214.77136	0.07
γ-Terpinene	C_10_H_16_	7.620112909	1,731,097.044	**8.52**
4-Thujanol	C_10_H_18_O	7.706679997	16,832.74405	0.08
Terpinolene	C_10_H_16_	7.890798398	34,496.08258	0.17
Linalool	C_10_H_18_O	7.954895716	71,394.31359	0.35
Borneol	C_10_H_18_O	8.578315076	212,615.7807	1.05
(−)-Terpinen-4-ol	C_10_H_18_O	8.656675878	135,369.6851	0.67
α-Terpineol	C_10_H_18_O	8.755613444	16,060.63109	0.08
*trans*-Dihydrocarvone	C_10_H_16_O	8.813361136	14,002.08286	0.07
2-isopropyl-4-methylanisole	C_11_H_16_O	9.131722127	346,801.9162	1.71
Thymol	C_10_H_14_O	9.458570918	1,535,481.091	**7.55**
Carvacrol	C_10_H_14_O	9.566596867	10,252,579.75	**50.44**
Caryophyllene	C_15_H_24_	10.48124424	179,293.3935	0.88
γ-Cadinene	C_15_H_24_	10.81288913	23,240.38415	0.11
β-Bisabolene	C_15_H_24_	10.96365994	142,573.3912	0.70
**Total identified compounds**				**97.16**
**Grouped Components (% *v*/*v*) of *O. vulgare* EO**				
**Classes of EO Constituents**		**%**		
Monoterpene hydrocarbons		33.43		
Oxygenated monoterpenes		62.04		
Sesquiterpene hydrocarbons		1.69		

**Table 2:** Antifungal activity of *O. vulgare* EO towards *M. perniciosa* strains; MIQ—minimal inhibitory quantity; MFQ—minimal fungicidal quantity. The results are presented as mean value ± standard deviation of three replicates, with the asterisks representing statistical significance, **** *p* < 0.0001, between the *O. vulgare* EO and Prochloraz-treated group.

Strain	Country of Strain Origin		*O. vulgare* EO (µL/disc)	Prochloraz-Mn (µL/disc)
MPS	Bosnia and Herzegovina	MIQ	0.10 ± 0.03 ****	5.00 ± 0.00
		MFQ	0.50 ± 0.00 ****	50.00 ± 0.00
MPH1	Netherlands	MIQ	0.30 ± 0.00 ****	5.00 ± 0.00
		MFQ	0.60 ± 0.00 ****	≥50.00
MPH2	Netherlands	MIQ	0.20 ± 0.00 ****	5.00 ± 0.00
		MFQ	0.80 ± 0.00 ****	50.00 ± 0.00
MPPS	Serbia	MIQ	1.00 ± 0.30 ****	5.00 ± 0.00
		MFQ	5.0 ± 0.00 ****	≥50.00
MPR	Serbia	MIQ	2.00 ± 0.00 ****	5.00 ± 0.00
		MFQ	4.00 ± 0.00 ****	≥50.00

**Table 3:** Yield performance of *Agaricus bisporus* fruiting bodies under different treatment conditions. Results are presented as the mean of three replicates ± SD, with the different letters in each row representing significant differences among the samples.

Sample	Treatment	Total Yield (kg/bag)	Healthy Fruiting Bodies (%)	Visual Grading *
1	Untreated control	1.19 ± 0.26 ^a^	100 ± 0 ^c^	5
2	Treated with prochloraz	1.21 ± 0.31 ^a^	100 ± 0 ^c^	5
3	Treated with 2% *O. vulgare* EO	1.24 ± 0.30 ^a^	100 ± 0 ^c^	5
4	Inoculated with *Mycogone perniciosa*	0.75 ± 0.12 ^a^	8 ± 2 ^a^	1
5	Inoculated with *M. perniciosa*, *O. vulgare* EO applied 4 h before inoculation	0.97 ± 0.16 ^a^	87 ± 9 ^b^	3
6	Simultaneously inoculated with *M. perniciosa* and treated with *O. vulgare* EO [34]	1.04 ± 0.19 ^a^	100 ± 0 ^c^	5

* Visual grading is on a scale of 1–5, with 1 being poor and 5 excellent.

## Data Availability

The original contributions presented in this study are included in the article. Further inquiries can be directed to the corresponding author.
